# ULTRASONOGRAPHIC EVALUATION OF BONE HEALING IN METACARPAL AND PHALANGEAL FRACTURES

**DOI:** 10.1590/1413-785220253306e285764

**Published:** 2025-11-10

**Authors:** Antonio Carlos da Costa, Thiago Barros Pinheiro, Anees Salim Saad, Fabio Hideki Nishi Eto, Yussef Ali Abdouni, Diego Figueira Falcochio

**Affiliations:** 1 Irmandade Santa Casa de Misericordia de Sao Paulo, Grupo de Mao e Microcirurgia, Sao Paulo, SP, Brazil.; 2 Irmandade Santa Casa de Misericordia de Sao Paulo, Cirurgia da Mao, Sao Paulo, SP, Brazil.; 3 Irmandade Santa Casa de Misericordia de Sao Paulo, Departamento de Ortopedia e Traumatologia Sao Paulo, SP, Brazil.

**Keywords:** Ultrasonography, Fracture Healing, Fracture, Metacarpals, Finger Phalanges, Ultrassonografia, Consolidação da Fratura, Fratura, Metacarpais, Falanges dos Dedos da Mão

## Abstract

**Objective:**

This study aimed to evaluate the use of ultrasonography (USG) compared to radiography in identifying callus formation and fracture healing in hand bones (metacarpals and phalanges).

**Methods:**

A prospective observational study was conducted with patients who sustained metacarpal and phalangeal fractures and were followed in the hand and microsurgery clinic of a philanthropic hospital in São Paulo between July 2023 and April 2024. Fractures were treated either conservatively or surgically with Kirschner wire fixation. Callus formation was monitored using serial weekly USG and radiographic examinations. Follow-up ended when bone healing was confirmed by both methods.

**Results:**

There was a difference in the mean time of callus appearance between ultrasonographic and radiographic evaluations for all analyzed variables.

**Conclusion:**

Ultrasonographic callus formation preceded radiographic callus appearance by approximately 18.2 days across all variables studied, suggesting that USG is a useful and alternative tool for the early diagnosis of bone healing in phalangeal and metacarpal fractures. ***Level of Evidence II; Prospective Observational Study***.

## RESUMO

**Objetivo:**

Esse trabalho teve como objetivo avaliar o uso da ultrassonografia (USG), comparativamente à radiografia, na identificação do calo ósseo e consolidação de fraturas da mão (ossos metacarpais e falanges).

**Método:**

Estudo observacional prospectivo dos pacientes com fratura dos metacarpais e falanges que foram acompanhados no ambulatório de cirurgia da mão e microcirurgia de um hospital filantrópico de São Paulo, no período entre julho de 2023 e abril de 2024, que foram tratados de forma clínica ou cirúrgica com fixação com fios de Kirschner. A formação do calo ósseo foi analisada através de exames de USG e radiografias seriadas semanalmente. O término do acompanhamento foi definido a partir da evidência de consolidação da fratura por ambos os exames.

**Resultados:**

Houve diferença entre o tempo médio do surgimento do calo ultrassonográfico em relação ao tempo médio de surgimento do calo radiográfico em todas as variáveis analisadas.

**Conclusão:**

O calo ultrassonográfico precedeu o calo radiográfico em aproximadamente 18,2 dias em todas as variáveis estudadas, elencando, dessa forma, a USG como uma ferramenta alternativa e útil para o diagnóstico precoce da consolidação óssea das fraturas das falanges e metacarpais. ***Nível de evidência II; Estudo Prospectivo Observacional***.

## INTRODUCTION

Hand fractures are among the most frequent in the human skeleton, with metacarpal and phalangeal fractures accounting for approximately 35% and 45% of all such injuries, respectively, predominantly affecting young adults.^1^ Early bone healing and functional recovery are the main objectives of treatment for these fractures.^2^

It is known that healing of these fractures occurs within three to four weeks,^3,4^ while clinical stability of the fracture occurs well before radiographic evidence of consolidation.^5^ Uncertainty and excessive reliance on an objective parameter, such as callus formation detected by radiography, for authorizing mobilization may lead to permanent stiffness of the joints around the fracture. This is because fractures with more than eight weeks of evolution, even without pain or mobility at the fracture site, may still not show unequivocal signs of consolidation on plain radiographs.^6^

Ultrasonography (USG) is capable of detecting callus formation and its progression earlier than radiography. Over the past decades, some studies have highlighted the importance of USG in the diagnosis of bone healing.^2,7,8^ This method is based on its ability to distinguish tissues with different densities. During the healing process, the periosteal soft callus grows, increases in density, and fills the fracture gap. This callus appears in various shades of gray depending on its density and can be distinguished from adjacent soft tissues.^9^

Despite this, we did not find studies in the literature that employed this method in the treatment of phalangeal and metacarpal fractures. Therefore, given the importance of early hand rehabilitation, the aim of this study was to evaluate the presence of bone callus by USG compared with radiography and to assess differences between phalangeal and metacarpal fractures, between closed and open fractures, and between conservative and surgical treatment.

## PATIENTS AND METHODS

We conducted a prospective observational study of patients with metacarpal and phalangeal fractures who were followed at our outpatient clinic between July 2023 and April 2024. Patients were treated either conservatively or surgically with Kirschner wire fixation. The study was approved by the Institutional Research Ethics Committee, in accordance with Resolution 196/96 (CAAE: 47826721.6.0000.5479).

A total of 32 patients were evaluated weekly until bone healing was confirmed by both methods.

The selected sample (Table 1) included patients over 18 years of age, of any sex, with acute fractures, open or closed, of any of the metacarpal or phalangeal bones of the hands. All patients were assessed by a single orthopedic surgeon experienced in USG, always using the same device.

**Table 1:** Description of all parameters evaluated in the patients.

Variable	Description
Age (years), mean ± SD	44.6 ± 14.6
**Sex, n (%)**	
Female	5 (26.3)
Male	14 (73.7)
**Fracture location, n (%)**	
Metacarpal (MC)	14 (73.7)
Phalanx	5 (26.3)
**Type of fracture, n (%)**	
Closed	15 (78.9)
Open	4 (21.1)
**Treatment, n (%)**	
Conservative	9 (47.4)
Surgical	10 (52.6)

Callus formation was analyzed through weekly USG and radiographic examinations, starting seven days after trauma in conservatively treated cases and, for those who underwent surgery, starting seven days after the surgical procedure. Follow-up ended when fracture consolidation was confirmed by both imaging methods.

The cutoff point for defining consolidation by USG was determined at the moment of identifying callus formation bridging at least two cortices of the studied bone. From the evidence of fracture consolidation on USG, patients were released from immobilization and, in operated cases, Kirschner wires were removed and rehabilitation was initiated. However, weekly follow-up was maintained until radiographic consolidation was identified.

For statistical analysis, qualitative characteristics of all patients were described using absolute and relative frequencies, and quantitative characteristics were described using summary measures (mean and standard deviation). Normality of distribution of callus formation times and the interval between methods was assessed using the Kolmogorov-Smirnov test, which did not indicate lack of normality in the data distribution.^10^

Times to callus formation with each assessment method were described and compared using the paired Student's t-test, with the interval between methods also presented.^10^ Both the times assessed with each method and the interval between methods were described according to the qualitative characteristics evaluated and compared across methods and categories of each characteristic using two-factor analysis of variance (ANOVA), with repeated measures between methods, followed by Bonferroni multiple comparisons to assess differences. Pearson correlations between age and times/interval were calculated to verify possible associations between callus formation times and patient age. Changes in correlations between methods and age were assessed using two-factor ANOVA.^11^

All analyses were performed using IBM SPSS for Windows, version 22.0, and data tabulation was performed using Microsoft Excel 2013. Tests were conducted with a significance level of 5%.

## RESULTS

Evaluation of the 32 patients demonstrated that bone callus formation on USG appeared earlier than on radiography.

Table 2 shows that the mean time to callus formation assessed by USG was 18.2 days shorter than the time assessed by radiography (p < 0.001).

**Table 2:** Description of bone callus formation times with each assessment method, comparison between methods, and the time interval between methods.

Variable	Mean ± SD	p
Callus USG	31.6 ± 9.5	<0.001
Callus X-ray	49.7 ± 11.2	
Δt	18.1 ± 6.2	

Paired Student´s t-test.

There was a difference between the mean time of ultrasonographic callus appearance compared with the mean time of radiographic callus appearance across all analyzed variables. No statistical influence was observed for characteristics such as sex, type of fracture, and treatment on the times or on the interval between times (p > 0.05). (Table 3)

**Table 3:** Description of bone callus formation times and the interval between methods according to the evaluated characteristics and the results of comparisons between categories.

Variable	Callus USG	Callus RX	Δt
**Fracture location**			
Metacarpal	34.8 ± 8.6	52.1 ± 10.9	17.3 ± 6.3
Phalange	22.8 ± 5.8	43 ± 10.4	20.2 ± 6.2
p	0.011	0.124	0.385
**Type of fracture**			
Closed	31.7 ± 10.5	48.5 ± 12.2	16.8 ± 5.7
Open	31.3 ± 5	54 ± 5.6	22.8 ± 6.7
p	0.931	0.403	0.090
**Treatment**			
Conservative	32.6 ± 12.3	51.3 ± 13.8	18.8 ± 6.1
Surgical	30.8 ± 6.7	48.2 ± 8.9	17.4 ± 6.6
p	0.699	0.559	0.644

Data expressed as mean ± SD; r: Pearson correlation; unpaired Student's t-test.

**Figure 1:**
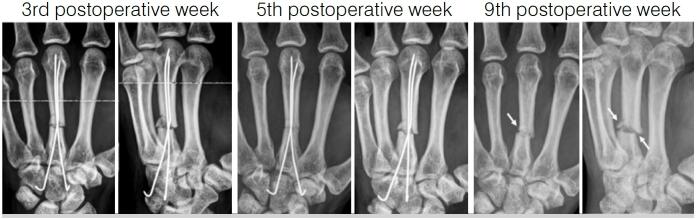
Postoperative follow-up radiographs of a metacarpal fracture. Kirschner wires were removed at the 5th postoperative week after evidence of ultrasonographic consolidation. Radiographic callus formation was observed only starting at the 9th postoperative week.

Source: Authors.

**Figure 2:**
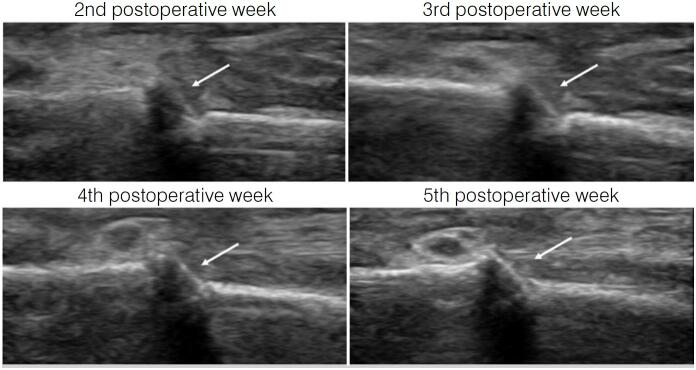
Serial postoperative ultrasonographic images demonstrating bone callus formation. At the 5th postoperative week, Kirschner wires were removed after visualization of the bone bridge at the fracture site.

Source: Authors.

Figures 1 and 2 illustrate a patient included in this study, with an open fracture of the third metacarpal that was surgically treated. After five postoperative weeks, when ultrasonographic callus was visualized, the Kirschner wires were removed. However, radiographic callus appeared only at the ninth postoperative week.

## DISCUSSION

Over the past three decades, several studies have attempted to demonstrate the importance of USG as a more accurate tool than conventional radiography for the early diagnosis of bone healing.^6,8,12-18^ This study was based on this prior knowledge, with the consideration that most of those series focused on long bones of the lower limbs, and no studies were found in the literature specifically addressing bone healing of hand fractures.

With the increasing use of USG by orthopedic surgeons and the advent of portable devices, examinations can be performed on an outpatient basis, providing more parameters for the management of hand fractures. USG has the advantages of being radiation-free, having lower operational costs, and enabling a more objective assessment of bone healing. This, in turn, helps to determine the ideal and safe time to begin patient rehabilitation,^13^ thereby reducing the uncertainties generated by radiographs as well as the risk of unfavorable outcomes secondary to imprudent treatment. Furthermore, the importance of early hand rehabilitation must be emphasized, since its joints are highly predisposed to stiffness following trauma and/or prolonged immobilization.^19^

Considering the results obtained in our case series, the time to callus formation detected by USG was on average 18.2 days earlier than that detected by radiography (p < 0.001), regardless of the variable analyzed, supporting the concept that ultrasonographic visualization of bone callus precedes radiographic detection. The standard deviation (SD) for both USG and radiographic callus diagnosis was relatively high because both phalanges and metacarpals were included in each group. As shown in Table 3, bone healing time for phalanges tends to be relatively shorter than for metacarpals.

Although factors such as fracture location (metacarpal or phalanx), fracture type (open or closed), and treatment method (conservative or surgical) did not demonstrate statistically significant differences due to an insufficient sample size, the differences observed in this study are consistent with those described in the literature when considering fracture location. However, when evaluating fracture type (open or closed), a discrepancy was noted.^20^ The mean healing time observed was approximately 4 weeks for metacarpals and 3 weeks for phalanges, which is consistent with expectations in the literature. In contrast, closed fractures in our series showed a longer mean healing time compared with open fractures, which likely reflects the still limited number of cases analyzed.

In our series, USG proved to be an alternative and useful tool for the early diagnosis of bone healing in phalangeal and metacarpal fractures.

## CONCLUSION

We conclude that ultrasonographic callus preceded radiographic callus by approximately 18 days across all studied variables, and that no differences were observed regarding fracture location, fracture type, or treatment modality.
